# A Novel Aqueous Micellar Two-Phase System Composed of Surfactant and Sorbitol for Purification of Pectinase Enzyme from *Psidium guajava* and Recycling Phase Components

**DOI:** 10.1155/2015/815413

**Published:** 2015-02-10

**Authors:** Mehrnoush Amid, Fara Syazana Murshid, Mohd Yazid Manap, Muhaini Hussin

**Affiliations:** ^1^Department of Food Technology, Faculty of Food Science and Technology, Universiti Putra Malaysia (UPM), 43400 Serdang, Selangor, Malaysia; ^2^Halal Products Research Institute, Universiti Putra Malaysia (UPM), 43400 Serdang, Selangor, Malaysia

## Abstract

A novel aqueous two-phase system composed of a surfactant and sorbitol was employed for the first time to purify pectinase from* Psidium guajava*. The influences of different parameters, including the type and concentration of the surfactant and the concentration and composition of the surfactant/sorbitol ratio, on the partitioning behavior and recovery of pectinase were investigated. Moreover, the effects of system pH and the crude load on purification fold and the yield of purified pectinase were studied. The experimental results indicated that the pectinase was partitioned into surfactant-rich top phase, and the impurities were partitioned into the sorbitol-rich bottom phase with the novel method involving an ATPS composed of 26% (w/w) Triton X-100 and 23% (w/w) sorbitol at 54.2% of the TLL crude load of 20% (w/w) at pH 6.0. The enzyme was successfully recovered by this method with a high purification factor of 15.2 and a yield of 98.3%, whereas the phase components were also recovered and recycled at rates above 96%. This study demonstrated that this novel ATPS method can be used as an efficient and economical alternative to the traditional ATPS for the purification and recovery of the valuable enzyme.

## 1. Introduction

Aqueous two-phase systems (ATPSs) are a potential industrial technology for the bioseparation of proteins and enzymes. The main advantages of ATPSs lie in their potential for upscaling, rapid mass transfer and phase equilibrium, possibility of continuous processing, and low energy requirements among other advantages [1]. ATPSs are typically formed by mixing two polymers, for example, polyethylene glycol (PEG) and dextran [2, 3] or PEG and maltodextrin [4], in an aqueous media or by mixing one polymer and one salt, such as PEG and a phosphate-based salt [5–7]. The main problem is that conventional ATPSs can be efficiently recycled efficiently, which results in high costs and environmental pollution. It has also been widely reported that additional, tedious operations, such as ultrafiltration, diafiltration, and crystallization, are needed to remove the phase-forming chemicals/polymers from the desired proteins that are recovered from conventional ATPSs [8]. To improve traditional ATPSs, a more economically and environmental friendly ATPS with the ability to retain the biological activities of enzymes is preferable to conventional ATPSs. A novel ATPS composed of a surfactant and sorbitol overcomes the drawbacks of the traditional ATPS method. This novel system enables the creation of two phases in which the surfactant-rich top phase and the sorbitol-rich bottom phase can be recycled with a high level of purified enzyme recovery [9]. Thus, in this manner, the novel ATPS method can minimize the overall cost by simplifying the process of the separation of the target proteins from the phase solution. Additionally, recycling the solution components can also minimize environmental pollution.

Guava (*Psidium guajava*) is an important commercial tropical fruits worldwide, and the production of this fruit is increasing due to high demand for guava as a healthy and nutritive table fruit [10]. The edible part of the guava, which composes 35–80% of the fresh fruit, is processed into many products, but the peel, which constitutes 15–20% of the whole fruit weight and possesses valuable types of enzymes, is not currently commercially utilized but rather discarded as waste material [11]. Guava peels can be used as a valuable, economical, and abundant source of media for the production of natural enzymes such as pectinase. To the best knowledge of the researchers, there is currently no information regarding the recovery of pectinase from guava peels using an ATPS composed of a surfactant and sorbitol. In the present study, the feasibility of recovering pectinase by recycling the phase components in a novel ATPS was investigated for the first time. The efficiency of partitioning pectinase in the ATPS and the effects of different types and concentrations of surfactants, including the concentration of sorbitol, the surfactant/sorbitol composition, the crude load and the pH, were investigated to achieve high a purification factor and high yield of the pectinase enzyme. Additionally, the recycling recovery of the surfactant and sorbitol were also investigated at each recycling step.

## 2. Materials and Methods

### 2.1. Materials

All chemicals and reagents were of analytical grade. Bradford reagent, 3,5-dinitrosalicylic acid (DNS), bovine serum albumin (BSA), and polygalacturonic acid were obtained from Sigma Chemical Co. (St. Louis, MO, USA). Triton X-100, Tween-80, sodium dodecyl sulphate (SDS), acetic acid, sodium citrate, citric acid, D-galacturonic acid, and sodium potassium tartrate (NaKC_4_H_4_O_6_·4H_2_O) were obtained from Merck (Darmstadt, Germany). Guava (*Psidium guajava*) fruits were purchased from local market (Selangor, Malaysia). Ripened guava fruits free of visual defects were selected based on the size uniformity at the same stage of ripening. The fruits were stored in a cold room at 4°C until use for the extraction procedure.

### 2.2. Extraction of Pectinase from Guava Peel

*Psidium guajava* fruit were washed with distilled water, peeled with a stainless steel knife, and cut into small pieces (1 mm × 1 mm × 3 mm). The pectinase extraction from the* Psidium guajava* peels with an ultrasound-assisted process was performed using an ultrasound device (Elma S 30 H, Elmasonic, Luckenwalde, Germany) with a piezoelectric transducer connected to a frequency generator (37 kHz). A Schott bottle was held in the ultrasound processor to extract the pectinase for 5 min at a 1 : 6 g/mL sample-to-solvent ratio with Tris-HCL buffer (pH 8.0) at room temperature. After extraction, the crude extracts were filtered and then centrifuged at 5,000 rpm for 10 min. The feedstock was maintained in a refrigerator at 4°C until use in ATPS experiment.

### 2.3. ATPS Composed of Nonionic Surfactants and Sorbitol

The systems for the purification of pectinase from* Psidium guajava* were prepared in graduated glass centrifuge tubes after weighing the appropriate amounts of each surfactant, the sorbitol, and the crude feedstock to reach a concentration of 20% (w/w) in the system. Deionized water was added to the mixture to achieve a final mass of 10 g. After complete mixing of all of the components for each mixture composition, each system was centrifuged at 4,000 g for 10 min. After the two phases became clear and transparent and the interface was well defined the bottom phase was carefully removed using a long needle syringe and a pipette was used to remove the top phase. The volumes of both the top and bottom phases were recorded. Subsequently, the samples were analyzed with a pectinase activity assay, and protein quantification was performed based on the Bradford analysis. Another tube with the same phase-forming components but without the feedstock was prepared as a blank to avoid interference.

### 2.4. Pectinase Activity Assay and Protein Concentration Determination

The reduction groups released from polygalacturonic acid as the substrate were determined to measure the activity of the extracted pectinase. The mixture of the extracted enzyme (0.5 mL) with polygalacturonic acid (0.5 mL) dissolved in 100 mM acetate buffer at pH 5.0 was incubated at 70°C for 60 min in a water bath. Then, 1 mL of DNS was added to the mixture to inhibit the reaction, and the sample was then placed in the boiling water for 5 min. Spectrophotometry (BioMate-3, Thermo Scientific, Alpha Numerix, Webster, NY, USA) was used to measure the released reducing sugar at 540 nm using galacturonic acid as the standard reducing sugar. One unit (U) of enzyme activity was defined as the amount of enzyme that catalyzed the release of 1 *μ*mol of polygalacturonic acid per minute [12]. The dye binding method was used to measure the protein contents of the samples as described by Bradford [13] using BSA as the standard.

### 2.5. Determination of the Enzyme Partitioning

The partition coefficient (*K*) of the pectinase was calculated as the ratio of the pectinase activity in the two phases:

(1) $$\begin{array}{c} K=\frac{A_{T}}{A_{B}}, \end{array}$$

where *A*_*T*_ and *A*_*B*_ are the pectinase activities in units/mL in the top phase and bottom phases, respectively.

The specific activity (SA) was defined as the ratio between the enzyme activity (U) in the phase sample and the total protein concentration (mg):

(2) $$\begin{array}{c} \text{SA}\left(\frac{\text{U}}{\text{mg}}\right)=\frac{\text{Enzyme}\;\;\text{activity}\;\left(\text{U}\right)}{\left[\text{Protein}\right]\;\left(\text{mg}\right)}. \end{array}$$

The selectivity (*S*) was defined as the ratio of the pectinase enzyme partition coefficient (*K*_*e*_) to the protein partition coefficient (*K*_*p*_):

(3) $$\begin{array}{c} S=\frac{K_{e}}{K_{p}}. \end{array}$$

The volume ratio (*V*_*R*_) was defined as the ratio of volume in the top phase (*V*_*T*_) to that in the bottom phase (*V*_*B*_):

(4) $$\begin{array}{c} V_{R}=\frac{V_{T}}{V_{B}}. \end{array}$$

The purification fold (*P*_FT_) was calculated as the ratio of the pectinase specific activity in the top phase to the initial pectinase specific activity in the crude extract [14]:

(5) $$\begin{array}{c} P_{\text{FT}}=\frac{\text{SA}\;\;\text{of}\;\;\text{phasesample}}{\text{SA}\;\;\text{of}\;\;\text{crudestock}}. \end{array}$$

Yield of pectinase in top phase was determined using

(6) $$\begin{array}{c} Y_{T}\;\;\left(\%\right)=\frac{100}{1+\left(1/{V_{R}}^{*}K\right)}, \end{array}$$

where *K* is partition coefficient and *V*_*R*_ is the volume ratio [14].

### 2.6. Morphological Study on Structure of Pectinase

The morphological properties of pectinase in presence of high and low concentrations of sorbitol were studied under a scanning electron microscope (SEM). The samples were attached to SEM tubes with 100 nm diameters using two-sided carbon tape. The samples were observed and examined at 2000x and 4000x magnifications. An acceleration potential of 20 kV was used to construct the micrographs [15].

### 2.7. Electrophoresis

Sodium dodecyl sulfate polyacrylamide gel electrophoresis (SDS-PAGE) with 6% stacking gel and 12% resolving gel was used to analyze the samples from crude extract, top and bottom phase in ATPS. Samples were diluted in a sample buffer and heated at 100°C for 5 min. Electrophoresis was run at 50 V and 12 mA for 1 h. The use of Coomassie brilliant blue R-250 staining method followed by destaining in a solution containing 40% (v/v) methanol and 10% (v/v) acetic acid allowed the detection and observation of desired protein bands [16].

### 2.8. Statistical Design and Analysis

All the experiments were organized using a completely randomized design with three replicates, repeated twice for reproducibility. Mean values of triplicate data for all the parameters were obtained and subjected to one-way analysis of variance (ANOVA). The statistical significance was accepted at *p* < 0.05 using Duncan's multiple range test (DMRT).

## 3. Results and Discussion

### 3.1. The Effect of Phase Components on Pectinase Stability

**Figure 1:**
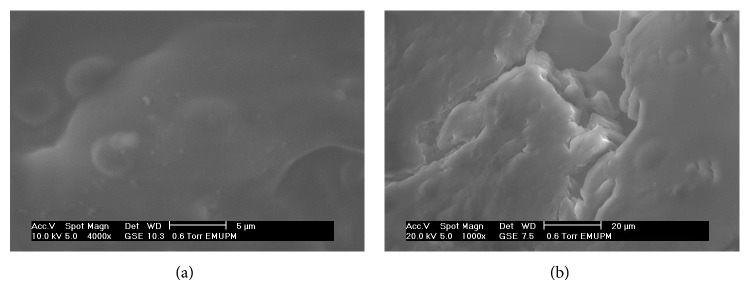
Effect of low concentration (20% w/w) (a) and high concentration (60% w/w) (b) of sorbitol on structure and morphology of pectinase enzyme.

Preliminary studies revealed that the enzyme was stable in Triton X-100, Tween 20, SDS, and sorbitol, which indicated that pectinase was suitable for the novel surfactant/sorbitol-based ATPS. Another study proved that surfactants promote the availability of reaction sites and increase the hydrolysis rate [17]. To determine the effects of each phase composition on the pectinase activity, crude feedstock pectinase was mixed with various compounds. The surfactant also induces enzyme activity and eventually reduces the rate of enzyme denaturation during hydrolysis [18] and thus functions as an inducer of enzyme activity by affecting the enzyme-substrate interaction. This process can prevent the inactivation of adsorbed enzymes, which directly facilitates the desorption of enzymes from the substrate [18–20]. High concentrations [60%] of Triton X-100 and Tween-80 slightly increased pectinase activity, whereas SDS partially inactivated this activity (Table 1). This latter effect was due to the binding of SDS, an ionic surfactant, to the proteins, which disrupted the majority of the globular proteins original structures. Moreover, the pectinase was mixed into sorbitol at different concentrations to determine its activity. In the presence of sorbitol, pectinase also exhibited high enzyme stability. The probable reason for this observation is that sorbitol helped to maintain the enzyme's open conformation by exposing the active site crevice surface and thus stimulating pectinase activity [18]. As shown in Table 1, a higher concentration of sorbitol (60%, w/w) inhibited the pectinase activity compared with the pectinase activity in 20% (w/w) sorbitol solution. Morphological observations revealed that, in 60% (w/w) sorbitol and pectinase, severe agglutination and rough surfaces within the interactions were observed, whereas 20% (w/w) sorbitol resulted in clear and smooth surfaces as shown in Figure 1. This observation might have resulted from the high concentration of sorbitol (60%, w/w) causing conformational changes in the pectinase structure that caused enzyme denaturation. Hence, this phenomenon decreased the pectinase activity and resulted in an incompatibility in the denatured enzyme-substrate interaction. Therefore, the authors speculate that the high concentrations of sorbitol denatured the pectinase and decreased its activity compared with the low concentration of sorbitol.

### 3.2. Selection of the Optimal Surfactants/Sorbitol ATPS for the Enzyme Partitioning

Table 2(a) shows the selectivity and purification factors of the pectinase from different types of surfactants and sorbitol. Notably, the effects of the surfactants on enzyme partitioning depended on the selective chemical interaction between the molecules, which was potentially influenced by the enzyme structure and chemical properties of the surfactant. The results shown in Table 2(a) indicate that the selectivity and purification factors of the pectinase in the nonionic surfactant/sorbitol system were significantly (*p* < 0.05) greater than those of ionic ATPS (Table 2(a)). According to some studies, this result might have been due to the protective amino acid surface loops, known as “lids” that shield the active site of the enzyme in the “closed” form [21, 22]. One study reported that the presence of nonionic surfactants causes the lid to undergo a conformational rearrangement that exposes the active site and forms the active “open” form of the enzyme [23]. This conformational change is probably a factor that is related to the greater activity and enzyme partitioning in the presence of nonionic surfactants. Accordingly, Triton X-100 increased enzyme partitioning compared with Tween-80, and SDS, which is an anionic surfactant, produced the smallest effect on enzyme partitioning. It has previously been suggested that ionic surfactant molecules bound to proteins might interrupt the tertiary structures of those proteins, and the interaction between ionic surfactants and proteins has been verified to be mediated via a combination of electrostatic and hydrophobic forces [24, 25]. Therefore, the surfactant head group plays a determining role in protein-surfactant interactions that preferentially begin with strong ionic bond formation between the surfactant polar groups [26]. This process would then inhibit enzyme partitioning in the system and eventually reduce the enzyme purification factor. The results revealed that the maximum achieved selectivity was 86.15 with a purification factor of 10.2 via the Triton X-100/sorbitol system; thus, this system was chosen for further optimization of the surfactant/sorbitol ATPS. Twenty systems were evaluated to optimize the pectinase partition efficiency in the Triton X-100/sorbitol system. Table 2(b) shows that the optimum condition for pectinase partitioning involved 26% (w/v) Triton X-100 and 23% (w/v) sorbitol, which resulted in purification factor of 11.71 and a yield of 91.2%. Based on this result, it can be deduced that pectinase partitioning performed well at low concentrations of surfactants and sorbitol. Notably, a high concentration of surfactants negatively affected the amount of solubilized enzyme and its catalytic action [27]. Similar effects were observed in sorbitol when the concentration was increased, and these effects were likely due to the gradual dehydration of the bottom phase as the concentration of nonionic surfactant in the top phase increased, which lead to an imbalance in pectinase retention in the top phase [28].

### 3.3. The Effect of Crude Feedstock Concentration and Total Volume of Aqueous Phase

**Figure 2:**
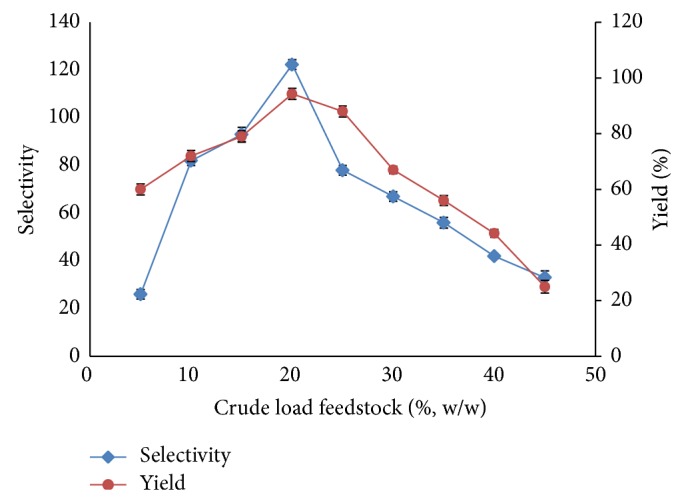
Effect of volume ratio on selectivity and yield of pectinase. The effect of volume ratio on pectinase partition behavior was investigated. The selectivity and yield were calculated as a function of the volume ratio, according to (3) and (6), respectively. The results were expressed as the mean of triplicate readings, which have an estimated error of ±10%.

The increase in crude load is an advantage in the recovery process of this ATPS technique. The effect of the loaded mass on enzyme partition is very crucial because it alters the phase-volume ratio [29] and the partition behavior of the target protein [30]. Additionally, high amounts of pectinase and contaminants in the system would cause decreases in ATPS performance. ATPS studies are performed by varying the crude loads, which can reach proportions of up to 50% (w/v).  Figure 2 illustrates the effect of crude load on pectinase recovery by showing the crude loads given 20% (w/v) of its maximum capacity per 10 g ATPS. Moreover, the selectivity and the yield of 20% (w/v) crude load ATPS were 122.3 and 94.3%, respectively. The composition and volume ratio of the ATPS were greatly reduced by the loading of large amounts of sample into the ATPS. The components in the crude stock were found to change the physical properties of the ATPS; hence, the ATPS could not be an optimum method for pectinase purification. This observation can be further explained when there is a highly accumulated precipitate at the interface that causes the loss of pectinase and pectinase contaminants during purification. Thus, these results clearly indicate that 20% sample loading is feasible when the top-phase recovery of pectinase from the crude extract occurs.

### 3.4. The Effect of System pH on Pectinase Partitioning

**Figure 3:**
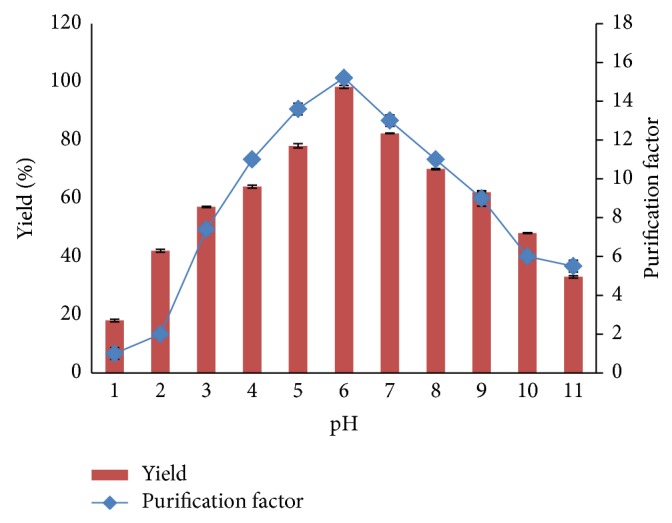
Influence of pH on yield and purification factor partitioning of pectinase in ATPS. Effect of various pH on the partitioning of pectinase in top phase was investigated. Triton X-100/sorbitol with 54.2% TLL and 20% crude load was used in this experiment. The purification factor and yield were determined according to (5) and (6), respectively.

The pectinase partitioning in ATPSs with different pHs are shown in Figure 3. Generally, biomolecule partitioning in an ATPS is influenced by the pH of the system, which affects the partitioning behavior of the protein by altering the charge of the target protein itself. Additionally, the manipulation of pH in an ATPS is correlated with the electrochemical interactions between the protein and solvent in the system [31]. Pectinase has an isoelectric point (pI) of 5.6; thus, at a pH of 6.0, it tends to be negatively charged, and partitioning depends on the surface properties more than the net charge. Pectinase is a negatively charged molecule and is thus favored to partition in the hydrophobic phase. However, the partitioning direction differed for the target enzyme, which tended to partition into the more enriched hydrophobic surfactant in the top phase. The changes in the partitioning behavior of pectinase were caused by protein charge. Basically, the purification factor and yield of the pectinase decreased at pHs above 6.0. This result was due to decreases in enzymatic activity at pHs above 6.0 because the enzyme is in an active state and stable in acidic pHs, and its activity might have decreased in the presence of neutral or alkaline pHs. Thus, the maximum purification factor was 15.2, and the enzyme yield was 98.3% at pH 6.0. A similar phenomenon was reported by Mohammadi and Omidinia [32] who purified recombinant phenylalanine dehydrogenase using an ATPS system. Hence, pH 6.0 was selected as the optimum pH for this study.

### 3.5. Recycling of Phase Components

An important advantage of this novel ATPS is that both of the phase-forming components could be recycled with high recovery percentages (5) because recovery rates greater than 96% could still be achieved in the fifth recycling run relative to the initial run (Table 3). Table 3 shows that there were only minor losses in the surfactant and sorbitol in the recycling steps; the recovery of the surfactant remained over 97% after five cycles relative to the initial amount. Therefore, the surfactant exhibited a good ability to transfer the desired protein into the top phase after several cycles of use. The surfactant reached its maximum capacity for accommodating negatively charged proteins, and thus the new protein that is dispensed into the ATPS can be partitioned into the surfactant-rich top phase [33]. This result also revealed that nearly 96% of the sorbitol was recovered during the recycling step, which indicates that the new system is economical and suitable for industrial applications and environmentally friendly.

### 3.6. Pectinase Recovery

**Figure 4:**
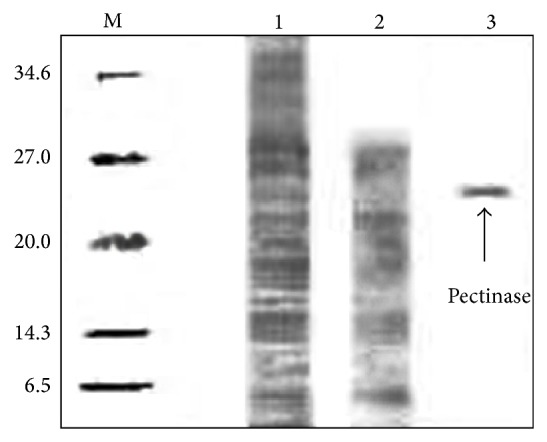
SDS-PAGE analysis on the recovery of pectinase. The protein molecular weight of the standard protein marker ranged from 6.5 to 34.6 kDa. Lines: M = protein molecular markers; 1 = crude feedstock; 2 = ATPS top phase lane; 3 = ATPS bottom phase.

The optimum condition of pectinase recovery which is obtained from ATPS consists of Triton X-100/sorbitol with TLL of 54.2% (w/v) and 20% (w/v) crude load at pH 6.0. By using 12% SDS-PAGE, the purity of pectinase from the guava peel is determined and recorded in Figure 4. Lane 1 shown in Figure 4 is referred to as crude feedstock with high amount of impurities bands. Lane 2 on the other hand contains aqueous phase sample which show lesser and fainter bands. The recovered sample from the top phase is identified as just one dark band with molecular weight of 24.4 kDa shown in lane 3. Thus, SDS-PAGE result represents the efficacy of the purification technique in this study in which giving maximum recovery of pectinase from* Psidium guajava*.

## 4. Conclusion

In the present study, the main factors that were evaluated were the effects of the type and concentration of surfactant, sorbitol concentration, TLL, crude load feedstock, and the pH of the pectinase. The optimum conditions obtained were 26% (w/v) Triton X-100 and 23% (w/v) sorbitol in combination with 54.2% TLL and 20% crude at pH 6.0. In the optimized condition, the enzyme yield was 98.3%, and the purification factor for the pectinase was 15.2. Therefore, this study demonstrated that the direct recovery of pectinase from guava waste via an ATPS based on a surfactant and sorbitol is a potential method for the purification of this enzyme from a fruit source. Notably, the pectinase purified from guava waste could have various industrial applications, including food processing in the production of fruits and beverages, olive oil extraction, and raw fiber treatment in the textile industry. Additionally, the purified pectinase will be employed in our biotechnology project in the near future. Moreover, the purified pectinase created with this novel method of purification (ATPS) provides opportunities for the use of other biological products, such as DNA, protein, and RNA, for recovery purposes. Notably, this novel method of ATPS is ecofriendly because it uses biodegradable surfactant and sorbitol for pectinase recovery. The highest recycling percentage that resulted from the use of these components was 96%, which indicates that this novel method of ATPS is a green technology that will help to promote a cleaner environment. This study established that the novel method of purification is an efficient and economical technology for pectinase recovery from fruit waste in large-scale production.

## Figures and Tables

**Table 1:** Effects of various phase compositions on the pectinase activity of* Psidium guajava*.

Phase composition	Concentration (%, w/w)	Pectinase activity
Triton X-100	20	100.1 ± 0.33^a^
	40	101.1 ± 0.02^a^
	60	120.4 ± 0.11^b^
	80	102.2 ± 0.01^c^

Tween-80	20	92.3 ± 0.11^a^
	40	91.1 ± 0.21^b^
	60	102.4 ± 0.22^c^
	80	98.6 ± 0.57^ab^

SDS	20	62.3 ± 0.32^a^
	40	71.2 ± 1.42^b^
	60	62.2 ± 0.09^a^
	80	61.1 ± 0.32^a^

Sorbitol	20	122.1 ± 0.07^a^
	40	108.6 ± 1.10^b^
	60	101 ± 0.22^ab^
	80	95.2 ± 0.52^c^

The sample sizes for all experiments were three. ^a–d^Mean values followed by different letters differ significantly (*p* < 0.05).

**(a):** 

System	Concentration of surfactant/sorbitol (% w/w)	TLL (%, w/w)	Selectivity	Purification factor
Triton X-100/sorbitol	16/19	53.1	61.11 ± 0.1^a^	3.21 ± 1.1^a^
	21/23	52.3	78.11 ± 1.1^b^	8.11 ± 1.1^b^
	25/26	54.2	86.15 ± 0.1^c^	10.2 ± 0.3^c^
	30/28	68.8	21.01 ± 1.1^d^	3.84 ± 0.2^d^

Tween-80/sorbitol	18/16	31.2	29.20 ± 1.1^e^	4.33 ± 1.1^ab^
	21/20	34.3	28.12 ± 0.2^e^	3.12 ± 1.1^e^
	23/23	41.2	22.11 ± 0.1^ed^	2.81 ± 0.1^d^
	28/24	44.3	18.11 ± 0.2^e^	3.62 ± 0.3^e^

SDS/sorbitol	14/12	32.1	3.23 ± 1.1^j^	1.12 ± 0.4^g^
	19/17	31.4	3.15 ± 1.3^k^	0.07 ± 0.2^h^
	23/20	44.2	2.11 ± 0.1^k^	0.26 ± 0.3^i^
	28/24	56.3	1.42 ± 0.1^jk^	0.02 ± 1.1^j^

**(b):** 

Triton X-100 (%, w/w)	Sorbitol (%, w/w)	Purification factor	Yield (%)
25	19	2.11 ± 0.2^a^	65.3 ± 0.5^a^
25	21	3.21 ± 1.1^b^	66.6 ± 0.2^b^
25	23	2.15 ± 0.1^c^	59.4 ± 1.1^c^
25	26	3.64 ± 0.2^d^	58.3 ± 0.3^ab^
25	28	3.58 ± 1.1^e^	73.3 ± 0.2^d^
26	19	9.11 ± 0.1^f^	82.6 ± 0.1^e^
26	21	8.13 ± 1.1^g^	83.4 ± 1.1^f^
26	23	11.42 ± 1.1^g^	91.2 ± 0.3^g^
26	26	8.11 ± 0.2^h^	78.3 ± 0.1^g^
26	28	7.72 ± 0.1^b^	77.6 ± 0.2^e^
27	19	4.83 ± 1.1^ab^	62.1 ± 1.1^f^
27	21	3.68 ± 0.1^i^	58.1 ± 0.2^g^
27	23	3.31 ± 0.2^i^	53.2 ± 0.3^h^
27	26	2.12 ± 0.1^i^	54.3 ± 0.4^i^
27	28	0.09 ± 0.2^i^	43.2 ± 1.1^j^
28	19	1.95 ± 0.1^j^	41.3 ± 0.1^k^
28	21	1.31 ± 1.1^i^	38.3 ± 0.3^jk^
28	23	2.82 ± 0.2^k^	32.6 ± 0.2^j^
28	26	1.11 ± 0.3^l^	28.4 ± 1.1^l^
28	28	0.02 ± 2.1^m^	24.3 ± 0.3^m^

The sample sizes for all experiments were three. ^a–m^Mean values followed by different letters differ significantly (*p* < 0.05).

**Table 3:** The recycles recovery of surfactant and sorbitol systems.

System	Initial	Recycle systems				
		First	Second	Third	Forth	Fifth
Recovery of surfactant (%)	99.2	99.1 ± 0.01	98.1 ± 0.2	97.6 ± 0.11	97.3 ± 0.03	97.1 ± 0.02
Recovery of sorbitol (%)	98.5	98.0 ± 0.02	97.3 ± 0.3	96.8 ± 0.02	96.3 ± 0.13	96.2 ± 0.01

## Abbreviations

ATPS: Aqueous two-phase system
BSA: Bovine serum albumin
DNS: Dinitrosalicylic acid
DMRT: Duncan's multiple range test
ANOVA: One-way analysis of variance
PEG: Polyethylene glycol
*P*_*F*_: Purification fold
SA: Specific activity
SDS-PAGE: Sodium dodecyl sulfate polyacrylamide gel electrophoresis
TLL: Tie line length.
